# Analysis of the Impact of Inactivated A71 Vaccine on the Incidence of Hand–Foot–Mouth Disease in Pudong New Area, Shanghai

**DOI:** 10.3390/vaccines12090962

**Published:** 2024-08-26

**Authors:** Dandan Yang, Wenmin Liu, Weiping Wang, Pengfei Deng, Chuchu Ye, Laibao Yang, Caoyi Xue

**Affiliations:** Shanghai Pudong New Area Center for Disease Control and Prevention, Shanghai 200136, China; yang163dd@163.com (D.Y.);

**Keywords:** inactivated A71 vaccine, vaccination, etiological surveillance, hand–foot–mouth disease

## Abstract

The aim of this study was to investigate the level of inactivated A71 (EV-A71) vaccination in Pudong New Area of Shanghai and its effects on the epidemiology and pathogen spectrum of hand–foot–mouth disease (HFMD) in this area, as well as to provide a basis for improving the prevention and control strategy of HFMD in this area. Data were collected from the “Comprehensive Management Cloud Platform for Immunization Program” system from December 2016 to December 2022. The data on HFMD cases from January 2012 to December 2022 were extracted from the “China Information System for Disease Control and Prevention”. A total of 484,056 doses were administered. The vaccination rate of the first dose was 14.03%, and the full vaccination rate was 13.33%. There were significant differences between the first dose and the full vaccination rate in different years (*χ*^2^ = 46,538.831, *p* < 0.001, *χ*^2^ = 50,013.946, *p* < 0.001). A total of 91625 cases of HFMD were reported, including 58 severe cases, and no deaths. Before and after the administration of the inactivated EV-A71 vaccine, there were statistical differences in the distribution of HFMD cases in terms of gender, household registration, occupation, and age (*p* < 0.001). The etiological surveillance results showed that the rate of enterovirus positivity was 84.15%, with 9.85% being EV-A71, 23.74% CV-A16, and 50.56% non-EV-A71 and non-CV-A16. The coverage rate of the inactivated EV-A71 vaccine in Pudong New Area was not high, and the incidence of HFMD showed a downward trend after the postmarketing of the vaccine. The majority of HFMD infections were non-EV-A71 and non-CV-A16, with CV-A6 accounting for the highest proportion. It is recommended to accelerate the development of combined vaccines to provide more antibody protection.

## 1. Introduction

Hand–foot–mouth disease (HFMD) is an acute infectious disease caused by a variety of pathogens, including enterovirus A71 (EV-A71) and the coxsackie virus (CV) types A6, A10, and A16. HFMD is mostly seen in children under 5 years old. In particular, EV-A71 is one of the main pathogens causing years of epidemics of HFMD [1]. It even causes severe illness and death through neurotropism and severe nervous system damage [2,3,4]. Before the use of the EV-A71 vaccine, 1489 clusters of HFMD cases were surveilled in Pudong New Area, Shanghai, from 2012 to 2016, and the result indicated that EV-A71 was predominant (67.42%) in 2011. Cox-A16 was the main type in 2012, 2015 and 2016, representing 50.40%, 49.61% and 70.00% of cases, respectively. From 2017 to 2021, the surveillance of 306 samples revealed that the Cox-A6 strain had become the dominant type, with a proportion of 79.89%.

The inactivated EV-A71 vaccine is a vaccine independently developed in China, and its immune effect has attracted great attention [5,6,7]. Pudong New Area, Shanghai, began to introduce the inactivated EV-A71 vaccine in December 2016. Children aged 6 months to 5 years can voluntarily receive the vaccine at their own expense. The full course of vaccination should include two doses, with an interval of 1 month between each dose.

This study analyzed the data on HFMD cases and etiological surveillance data before and after the introduction of the inactivated EV-A71 vaccine in Pudong New Area, as well as the vaccination level in conjunction with the vaccination situation, to further understand the efficacy of the inactivated EV-A71 vaccine. The findings provide a basis for prevention and control strategies for HFMD in this district.

## 2. Materials and Methods

### 2.1. Data Sources

Vaccination data for the inactivated EV-A71 vaccine in Pudong New Area, from 1 December 2016 to 31 December 2022, were extracted from the “Comprehensive Management Cloud Platform for Immunization Program” system, and the population data of children aged 6 months to 5 years during the same period were obtained. The data on HFMD cases, with their current residential addresses in the Pudong New Area between 1 January 2012 and 31 December 2022, were extracted from the “China Information System for Disease Control and Prevention”. The population data were obtained from the Shanghai Statistical Yearbook. The samples for etiological surveillance were collected from sentinel surveillance hospitals for HFMD in the Pudong New Area from 2012 to 2022.

### 2.2. Sample Collection and Pathogen Detection

Samples were collected from the children in accordance with the “Shanghai Hand, Foot and Mouth Disease Surveillance Program” (version 2016). Collect cases with fever accompanied by rashes on the hands, feet, mouth, and buttocks; some cases may not have fever. Collect throat swabs, fecal samples, and blister fluid from the cases. Reverse transcription–polymerase chain reaction was used for viral nucleic acid detection. After the nucleic acid test was confirmed positive, typing of EV-A71, CV-A16, and CV-A6 was performed [4]. HFMD-enhanced surveillance sentinel hospitals collected at least three specimens of common HFMD cases per week during the epidemic period (3–10 months) and at least eight specimens per week during the nonepidemic period (1–2 months, 11–12 months). If the sampling requirements could not be met, all patients were sampled, and all specimens were sent to the District Center for Disease Control and Prevention within 48 h after collection for enterovirus-related pathogen testing.

### 2.3. Main Indicators

Inactivated EV-A71 vaccine vaccination rate (%) = actual number of each shot/number of children 6 months to 5 years old that should be vaccinated × 100%.

Assuming that each child received an average of two doses, the estimated vaccination coverage of inactivated EV-A71 vaccine = (inactivated EV-A71 vaccine doses/2)/number of children 6 months to 5 years old that should be vaccinated [8,9,10].

Positive pathogen detection rate (%) = number of positive pathogen detection cases/total number of tests × 100%.

Estimated incidence of HFMD caused by EV-A71 infection = (positive EV-A71 rate × number of common cases of HFMD + number of severe and fatal cases of HFMD caused by EV-A71 infection)/100,000 people [11].

Definition of time before and after vaccine application: Vaccination with the inactivated EV-A71 vaccine began in Pudong New Area in December 2016. The period 2012–2016 was defined as the premarketing period of the vaccine, and the period 2017–2022 was defined as the postmarketing period of the vaccine.

### 2.4. Data Analysis

The characteristics of patients who received the inactivated EV-A71 vaccine were analyzed using descriptive epidemiological methods. Excel 2016 and SPSS 24.0 software were used for the statistical analysis. The trend Chi-square test was used to analyze the change in vaccination rate over time, and the difference in rates was analyzed using the Chi-square test. A *p* value of <0.05 was considered statistically significant.

## 3. Results

### 3.1. Analysis of Inactivated EV-A71 Vaccination

#### 3.1.1. Vaccination Overview

**Figure 1:**
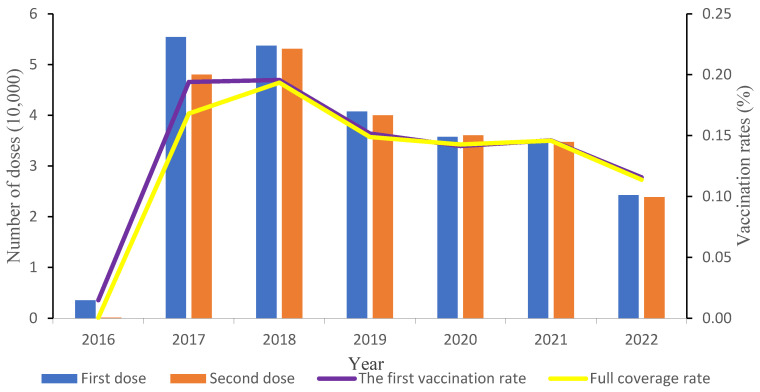
Distribution of inactivated EV-A71 vaccination in Pudong New Area, 2016–2022.

From December 2016 to December 2022, a total of 484,056 patients were vaccinated: 250,953 males (51.8%) and 233,103 females (48.2%). Children living in Pudong New Area received 239,195 doses (49.4%), and migrant children received 244,861 doses (50.6%). The first doses amounted to 248,201 shots, for a vaccination rate of 14.03%. The total number of shots was 235,855, for a vaccination rate of 13.33% (235,855/1,769,260). The first dose and full vaccination rates in 2018 (19.57% and 19.35%, respectively) represented the inflection point, around which there was a trend of first increasing and then decreasing. The rates of the first dose and the full range of vaccination were significantly different in different years (*χ*^2^ = 46,538.831, *p* < 0.001; *χ*^2^ = 50,013.946, *p* < 0.001) (Figure 1).

**Figure 2:**
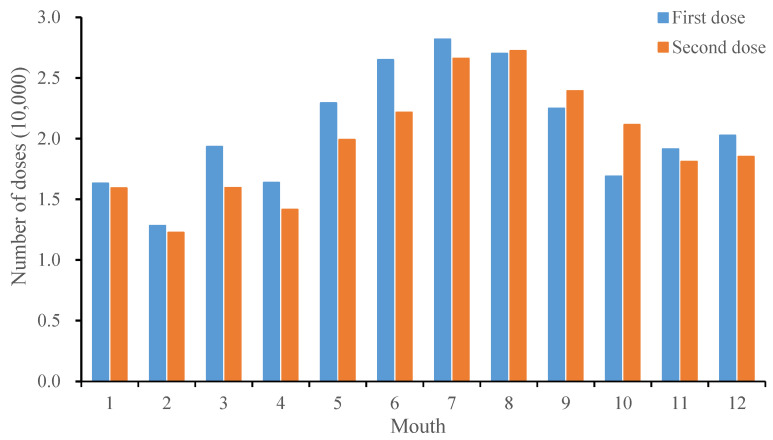
Distribution of inactivated EV-A71 vaccination by mouth in Pudong New Area, 2016–2022.

June–September was the peak of vaccination, accounting for 42.16% of the total vaccination volume, with the most first does administered in July (28,000 doses) and the most second doses administered in August (27,000 doses) (Figure 2).

#### 3.1.2. Estimation of Vaccination Coverage

The average estimated vaccination rate of the inactivated EV-A71 vaccine in Pudong New Area from 2016 to 2022 was 13.78%, with the highest estimated vaccination rate in 2018 and the lowest estimated vaccination rate in 2016. The difference in the estimated vaccination rate in different years was statistically significant (*χ*^2^*_for trend_* = 43,734.461, *p* < 0.001) (Table 1).

### 3.2. Analysis of the Incidence of HFMD in the Pudong New Area before and after the Use of the Inactivated EV-A71 Vaccine

#### 3.2.1. Changes in the Incidence of HFMD before and after Vaccination

From 2012 to 2022, a total of 91,625 cases of HFMD were reported, including 58 severe cases. There were no deaths. The average annual reported incidence rate fluctuated between 10.72/100,000 and 296.74/100,000. From 2012 to 2016, the average incidence rate was 232.38/100,000, showing an “alternate bearing” distribution; that is, the incidence peaked every other year. From 2017 to 2022, the average incidence rate was 84.56/100,000, with the incidence peaking in 2018 (221.43/100,000). The incidence rate was lower in subsequent years (*χ*^2^*_for trend_* = 35,505.164, *p* < 0.001) (Table 2).

#### 3.2.2. Changes in the Epidemiological Characteristics of HFMD before and after Vaccination

A total of 54,766 male HFMD patients and 36,859 female patients were reported, for a male-to-female ratio of 1.49:1. The age of onset ranged from 0 to 67 years, with 81.52% of the patients being 0–5 years old (74,692/91,615) and 23.95% being 1 year old (21,944/91,625). Children in informal care and nursery children together accounted for 91.45%, with children in informal care children alone accounting for 53.45%. There were significant differences in sex, household registration, occupation, and age distribution from before to after vaccine availability (all *p* < 0.001) (Table 3).

**Figure 3:**
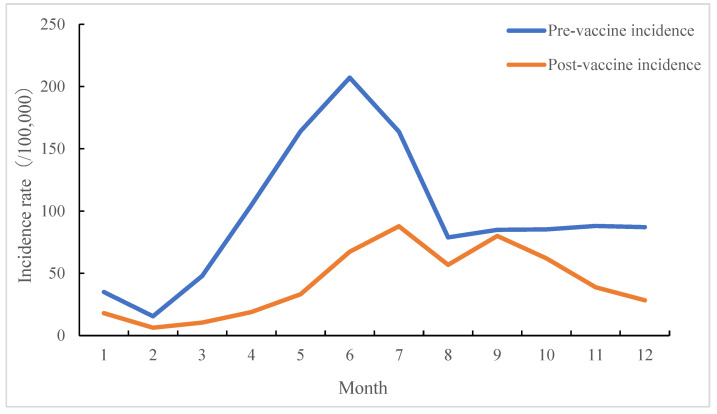
HFMD incidence by month before and after administration of inactivated EV-A71 vaccine.

The incidence of HFMD can be found throughout the year, with the peak occurring from May to July, the highest rate occurring in June (207.23/100,000), and the lowest rate occurring in February (6.27/100,000). The incidence of HFMD before the availability of the inactivated EV-A71 vaccine fluctuated significantly, reaching a peak in June (207.23/100,000) and then decreasing rapidly in August (78.78/100,000). The incidence rate from September to December was 85.28/100,000. After the availability of the inactivated EV-A71 vaccine, the overall incidence rate decreased in the whole district, the incidence rate increasing from June to September, with two small incidence peaks occurring in July (87.75/100,000) and September (80.01/100,000). The incidence rate was lower in other months. The incidence of HFMD in a given month differed between before and after the availability of the inactivated EV-A71 vaccine (*χ*^2^ = 88.570, *p* < 0.001) (Figure 3).

#### 3.2.3. Etiological Surveillance of HFMD before and after Vaccination

**Figure 4:**
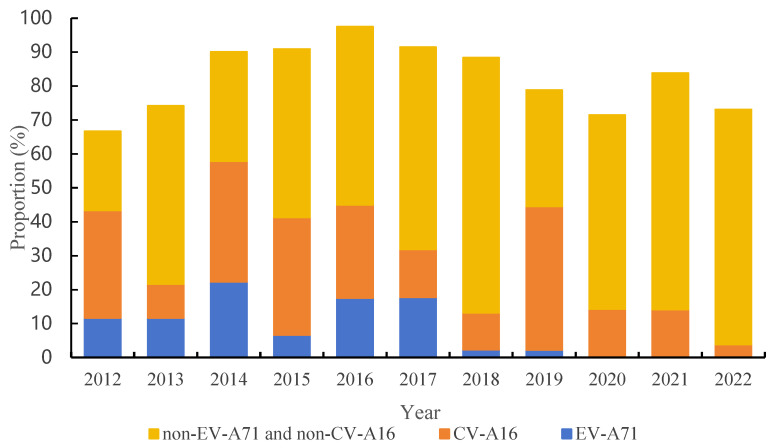
Etiological surveillance results of HFMD before and after vaccination of inactivated EV-A71 vaccine in Pudong New Area, 2012–2022.

During the 2012–2022 sentinel surveillance of HFMD etiology in the Pudong New Area, a total of 1167 HFMD patient samples were collected. The rate of enterovirus positivity was 84.15% (982/1167). Among the positive samples for HFMD etiology monitoring before the availability of the inactivated EV-A71 vaccine, 13.95% were positive for EV-A71, and the proportion decreased to 5.49% after availability. For HFMD, the years when the dominant viral strain was EV-A71 were 2014 and 2016–2017; the years when the dominant viral strain was CV-A16 were 2014, 2015, and 2019; and the years when the dominant viral strains were non-EV-A71 and non-CV-A16 were 2013, 2016–2018, and 2020–2022. The differences in pathogenic composition between the different strains of EV-A71, CV-A16, and non-EV-A71 and non-CV-A16 in each year were significant (*χ*^2^ = 203.253, *p* < 0.001) (Table 4 and Figure 4).

## 4. Discussion

From 2016 to 2022, a total of 484,056 doses of the inactivated EV-A71 vaccine were administered in the Pudong New Area. The vaccination rate for the first dose was 14.03%, the full course was 13.33%. The vaccination rates first increased and then decreased, which is consistent with the Jinshan District of Shanghai [11] and Jining City [8]. The administration rate of the inactivated EV-A71 vaccine was low when the vaccine was first introduced for use in December 2016, but the administration of the inactivated EV-A71 vaccine gradually increased in 2017, indicating that parents were gradually becoming more accepting of this vaccine. The reason may be that media reports, various publicity channels, information dissemination on science popularization platforms, and the societal information exchange increased parents’ awareness of the vaccine [12,13]. Therefore, timely disclosure of vaccination information, strengthening official science popularization, and correctly guiding parents to gain knowledge on vaccines are key to improving vaccination coverage.

The data from this study showed that most vaccinations occurred between June and September, which was similar to the high-vaccination months reported in Zhejiang Province [14]. This finding is basically consistent with the peak incidence of HFMD [9], which reveals that fluctuations in the incidence of infectious diseases have a positive effect on vaccination. However, from the perspective of prevention, antibodies are usually produced for several weeks after vaccination, before they reach their peak in the body’s circulation [15]. All vaccines should be administered 2–3 months before the peak of the infectious disease. Therefore, early planning and implementation of vaccination promotion before the annual HFMD epidemic peak can fully support the primary prevention role of the vaccine and better control the incidence of infectious diseases.

The basic reproductive number of the EV-A71 virus *R*_0_ is 5.5 [16] (quartile: 4.2–6.5), which means that, in theory, the vaccination rate required to interrupt transmission is 91% (85–94%). In this study, the estimated overall vaccination rate of the inactivated EV-A71 vaccine was 13.78%, with the highest rate of 19.46% in 2018, which is far lower than the vaccination rate required to interrupt transmission. The estimated vaccination rates in different areas are different; for example, the estimated rates in Guangdong Province in 2016 and 2017 were 2.67% and 10.07% [9], respectively; the estimated 2016–2019 vaccination rate in Zhaoqing city was 6.8% [17]; the estimated 2016–2021 vaccination rate in Jining city was 7.98% [8]. In addition, the estimated vaccination rate varies from city to city in Sichuan Province: Chengdu city boasted 29.15%, while Ganzi Prefecture reported only 0.79% [18]. This suggests that the publicity intensity of inactivated EV-A71 vaccine, policy promotion, the level of socioeconomic development, public concern, and willingness to vaccinate have great impacts on the administration of vaccines which are not covered in immunization programs.

From 2012 to 2022, a total of 91,625 HFMD cases were reported in the Pudong New Area, which has implemented inactivated EV-A71 vaccination since December 2016. Before the availability of the vaccine, i.e., between 2012 and 2016, the average incidence rate was 232.38/100,000, which was slightly lower than that in the Minhang District of Shanghai (290.09/100,000) [19] and slightly higher than that in Jinshan District of Shanghai (179.66/100,000) [11]. The incidence showed a distribution of “alternate bearing”, which is similar to that in Minhang District of Shanghai [19]. In Kunming city, Yunnan Province [20], Puyang city, Henan Province [21], and other places, the incidence rates were consistent, which was in line with the statement that the epidemic interval of HFMD was 2–3 years, as mentioned in the “Guidelines for the Prevention and Control of HFMD (2008 Edition)” issued by the Ministry of Health in 2008 [11,22]. After the vaccine was used in the Pudong New Area in 2017–2022, the average incidence rate was 84.56/100,000, and the overall incidence rate showed a downward trend. The peak incidence occurred in 2018, but the incidence in the other years was low, indicating that after the use of the EV-A71 vaccine, the proportion of severe cases of HFMD decreased significantly, indicating that vaccination with the inactivated EV-A71 vaccine can prevent severe cases of EV-A71 infection, consistent with other studies [9,10,23,24].

According to the etiological surveillance data of HFMD, the male-to-female ratio of onset was 1.49:1, and the age range of onset was wide, though more than 81.52% were children under 5 years old, and 91.45% were children in informal care and nursery children, which was similar to the results of a study conducted in Gansu Province [25]. The possible reasons are that boys are more active, have more opportunities to be exposed to pathogens, and because their immune system is not yet fully developed at a young age, resulting in a higher incidence of various diseases.

The incidence of HFMD shows obvious seasonal characteristics, with double peaks in summer and autumn, and the climatic factors are closely related to the incidence of HFMD [10], suggesting that climatic factors should be considered when studying and assessing the occurrence of HFMD and that disease guidance and emergency treatment in places with a high incidence of cluster events, such as childcare institutions, should be strengthened. Moreover, during the development of various health science popularization activities for HFMD, the integration of knowledge on meteorological factors, vaccines, and disease should be strengthened to enhance the effect of science popularization.

The main pathogens causing HFMD were non-EV-A71 and non-CV-A16 enteroviruses, followed by the CV-A16 enterovirus. During 2012–2016, the EV-A71 enterovirus accounted for 13.95%, the CV-A16 virus accounted for 27.91%, while non-EV-A71 and non-CV-A16 enteroviruses accounted for 42.03% of the cases. In 2017–2022, the EV-A71 enterovirus accounted for 5.49%, the CV-A16 virus accounted for 19.29%, while the non-EV-A71 and non-CV-A16 enteroviruses accounted for 59.65% of the cases, suggesting that the inactivated EV-A71 vaccine can reduce the risk of EV-A71 enterovirus infection but does not have protection against HFMD caused by other enterovirus infections [4], as it offers no cross-protection against other viruses. Some studies have shown that EV-A71 vaccination may not affect the prevalence of HFMD until it reaches a certain level of vaccine coverage for 2 to 3 years. After the start of vaccination in December 2016, the percentage of HFMD cases caused by the EV-A71 enterovirus in 2018 decreased significantly to 2.33%. However, in the same year, the detection of non-EV-A71 and non-CV-A16 enteroviruses accounted for 75.19% of the cases, with CV-A6 accounting for 90.72% (88/97) of the cases. From 2015 to 2022, the results of pathogen surveillance of HFMD showed that CV-A6 accounted for more than 82% of the HFMD cases caused by non-EV-A71 and non-CV-A16 enteroviruses. China CDC weekly [26] suggested that CV-A6 has become the main pathogen of severe cases of HFMD under 5 years old, similar to the studies on the HFMD virus in Puyang, Henan Province [21], Nanning City [27], and provincial HFMD monitoring network laboratories in China [28]. These results illustrate the changing trend of the pathogenic spectrum of HFMD in China in recent years and suggest that the HFMD cases should be more accurately monitored, and a combination vaccine should be developed to prevent HFMD caused by different viruses. Reducing the overall incidence of HFMD is also very important.

This study also has certain limitations. From 2020 to 2022, the country was in a major epidemic prevention and control period. On one hand, the public’s knowledge of infectious disease prevention and control increased, and there was more attention to information related to infectious diseases. On the other hand, schools and childcare institutions, which are prone to cluster events, paid more attention to morning checks and disinfection. The implementation of these measures is conducive to preventing the occurrence and spread of respiratory infectious diseases.

## 5. Conclusions

In summary, after the availability of the inactivated EV-A71 vaccine, the incidence and severity of HFMD caused by EV-A71 infection in the Pudong New Area were lower, but vaccination coverage is still not universal, and the estimated vaccination rate is low. Moreover, departments should increase vaccination publicity, specify vaccination safety measures, and establish herd immunity to reduce the risk of infection with EV-A71 of susceptible populations. Health education about HFMD and its vaccine can be strengthened through vaccination classes for parents, health education courses at schools/childcare institutions, and official WeChat accounts to promote preventive knowledge among parents of children to receive non-National Immunization Program (non-NIP) vaccines, such as the inactivated EV-A71 vaccine. In addition, the research and development of HFMD combination vaccines should be accelerated to prevent infection by other dominant strains, such as CV-A16 and CV-A6, to reduce the risk of HFMD in children.

## Figures and Tables

**Table 1:** Vaccination of inactivated EV-A71 vaccine in Pudong New Area, 2016–2022.

Year	Dose (n)	Number of School-Age Children (n)	Estimated Vaccination Rate (%)
2016	3642	239,755	1.52
2017	103,452	285,714	18.10
2018	106,798	274,436	19.46
2019	80,738	268,672	15.03
2020	71,819	252,868	14.20
2021	69,516	238,173	14.59
2022	48,091	209,642	11.47
Total	484,056	1,769,260	13.78
*χ* ^2^ *_for trend_*	43,734.461		
*p*	<0.001		

**Table 2:** Incidence of HFMD in Pudong New Area, 2012–2022.

Year	Cases (n)	Incidence Rate (/100,000)	Severe Cases (n)	Proportion of Severe (%)
2012	13,152	249.85	17	0.13
2013	10,093	186.60	5	0.05
2014	16,176	296.74	17	0.11
2015	10,722	195.84	6	0.06
2016	12,832	233.27	7	0.05
2017	5258	95.11	2	0.04
2018	12,290	221.43	1	0.01
2019	5447	97.84	2	0.04
2020	1322	23.25	1	0.08
2021	3713	64.38	0	0.00
2022	620	10.72	0	0.00
Total	91,625	150.25	58	0.06
*χ* ^2^ *_for trend_*		35,505.164		78.492
*p*		<0.001		<0.001

**Table 3:** Incidence and proportion of HFMD in different populations before and after vaccination of inactivated EV-A71 vaccine in Pudong New Area.

Characteristics	Cases n (%)	Cases of Pre-Vaccine n (%)	Cases of Post-Vaccine n (%)	*χ* ^2^	*p*
Sex				13.269	<0.001
Male	54,766 (59.77)	37,892 (60.17)	16,874 (58.90)		
Female	36,859 (40.23)	25,083 (39.83)	11,776 (41.10)		
Household registration				706.224	<0.001
Local	54,729 (59.73)	35,787 (56.83)	18,942 (66.12)		
Nonlocal	36,896 (40.27)	27,188 (43.17)	9708 (33.88)		
Occupation				1164.169	<0.001
Children in informal care	48,969 (53.45)	34,559 (54.88)	14,410 (50.30)		
Nursery children	34,815 (38.00)	24,363 (38.69)	10,452 (36.48)		
Students	7297 (7.96)	3787 (6.01)	3510 (12.25)		
Other	544 (0.59)	266 (0.42)	278 (0.97)		
Age (year)				1014.390	<0.001
0~<1	5740 (6.26)	3931 (6.24)	1809 (6.31)		
1~<2	21,944 (23.95)	15,364 (24.40)	6580 (22.97)		
2~<3	16,908 (18.45)	12,308 (19.54)	4600 (16.06)		
3~<4	18,176 (19.84)	12,921 (20.52)	5255 (18.34)		
4~<5	11,924 (13.01)	8276 (13.14)	3648 (12.73)		
5~<6	6496 (7.09)	4348 (6.90)	2148 (7.50)		
≥6	10,437 (11.39)	5827 (9.25)	4610 (16.09)		
Total	91,625 (100)	62,975 (100.00)	28,650 (100.00)		

**Table 4:** Etiological surveillance results of HFMD before and after administration of inactivated EV-A71 vaccine in Pudong New Area, 2012–2022.

	EV-A71		CV-A16		Non-EV-A71 and Non-CV-A16		CV-A6 in Non-EV-A71 and Non-CV-A16	
Year *	Cases (n)	Proportion (%)	Cases (n)	Proportion (%)	Cases (n)	Proportion (%)	Cases (n)	Proportion (%)
Pre-vaccine	84	13.95	168	27.91	253	42.03	140	55.34
2012	14	11.67	38	31.67	28	23.33	0	0.00
2013	14	11.67	12	10.00	63	52.50	3	4.76
2014	27	22.31	43	35.54	39	32.23	27	69.23
2015	8	6.61	42	34.71	60	49.59	54	90.00
2016	21	17.50	33	27.50	63	52.50	56	88.89
Post-vaccine	31	5.49	109	19.29	337	59.65	300	89.02
2017	25	17.73	20	14.18	84	59.57	77	91.67
2018	3	2.33	14	10.85	97	75.19	88	90.72
2019	3	2.19	58	42.34	47	34.31	39	82.98
2020	0	0.00	1	14.29	4	57.14	4	100.00
2021	0	0.00	14	14.14	69	69.70	57	82.61
2022	0	0.00	2	3.85	36	69.23	35	97.22
Total	115	9.85	277	23.74	590	50.56	440	74.58
*χ* ^2^	203.253							
*p*	<0.001							

*: HFMD sampling was carried out in some months because the country was in a major epidemic prevention and control period from 2020 to 2022.

## Data Availability

Restrictions apply to the availability of these data, which were collected from the Shanghai Pudong and National Information System. Data are available from the authors with permission from the Shanghai Pudong New Area Center for Disease Control and Prevention.
